# Neuroinvasive West Nile Virus Disease in an Elderly Patient with Diffuse Large B-Cell Lymphoma Treated with R-CHOP Therapy: A Case Report

**DOI:** 10.4274/balkanmedj.galenos.2019.2018.12.74

**Published:** 2019-08-22

**Authors:** Barış Arslan, Hasan Murat Gündüz, Nurdan Ünlü, Gökhan Çavuş, Dilek Menemenlioğlu

**Affiliations:** 1 Clinic of Anesthesia and Intensive Care, Adana City Training and Research Hospital, Adana, Turkey; 2 Department of Anesthesia and Intensive Care, Çukurova University School of Medicine, Adana, Turkey; 3 Department of Neurosurgery, Adana City Training and Research Hospital, Adana, Turkey; 4 Medical Microbiology Specialist, Public Health General Directorate, Microbiology Reference Laboratories and Biological Products Department, National Arboviruses and Viral Zoonoses Laboratory, Ankara, Turkey

**Keywords:** Lymhoma, Turkey, West Nile virus

## Abstract

**Background:**

West Nile virus is an arthropod-borne virus (arbovirus) and emerging cause of significant illness in European and Mediterranean countries. West Nile virus infection can cause severe and potentially fatal neurological illnesses, including encephalitis, meningitis, and acute flaccid paralysis. Additionally, immunosuppression, alcohol abuse, old age, and diabetes mellitus are common factors associated with West Nile neuroinvasive disease.

**Case Report:**

In August 2018, a 60-year-old male patient with a history of diffuse large B-cell lymphoma initially presented with symptoms including abdominal pain and distention, nausea, and vomiting. Three days after open abdominal surgery due to adhesive small bowel obstruction, he developed fever, prominent tremors, and rapidly progressing flaccid paralysis. The identification of West Nile virus RNA in the serum sample led to the diagnosis of West Nile neuroinvasive disease.

**Conclusion:**

Clinicians should evaluate patients with acute flaccid paralysis for the evidence of West Nile neuroinvasive disease. It is particularly important for healthcare providers to consider West Nile neuroinvasive disease in the differential diagnosis of aseptic meningitis, encephalitis, and acute paralysis cases, especially in endemic areas.

West Nile virus is a single-stranded RNA virus that was first isolated from the blood of a woman suffering from fever in Uganda in 1937 (1). Transmission of West Nile virus occurs primarily through bites of infected mosquitoes. In a very small number of cases, West Nile virus has also been found to be transmitted through blood transfusions, organ transplants, breastfeeding, or intrauterine exposure (2). The infection is generally asymptomatic but presents as a mild self-limiting febrile illness in 20% of patients (3). Meningitis, encephalitis, and flaccid paralysis or a combination of these occur in <1% of West Nile virus-infected patients.

Advanced age, immunosuppression, alcoholism, and diabetes mellitus are associated with severe West Nile neuroinvasive disease (3). Herein, we report a case of West Nile neuroinvasive disease in an elderly immunocompromised patient presenting with encephalitis and flaccid paralysis. Written informed consent was obtained from the patient’s family members for publication.

## CASE PRESENTATION

A 60-year-old man was diagnosed with diffuse large B-cell lymphoma. He had previously received three cycles of rituximab, cyclophosphamide, hydroxydaunorubicin, vincristine, and prednisone; however, the fourth cycle was canceled due to neutropenia. After 3 weeks, he was admitted to the surgical ward with the primary complaints of abdominal pain, vomiting, nausea, and abdominal distension. On the same day, he was diagnosed with ileus and he immediately underwent surgery. On the 3^rd^ hospital day, he developed a persistent fever and rapidly progressing muscle weakness. The patient was then transferred to the intensive care unit.

Upon physical examination, he was conscious and his pupils were bilaterally reactive to light; however, deep tendon reflexes were absent in all four of his limbs. The power in his upper limbs was 2/5, and that in his lower limbs was 0/5, but there was no sensory loss. His pulse was 110/min, blood pressure was 130/70 mm Hg, and body temperature was 37.5 °C. His oxygen saturation was 92% with oxygen therapy. Subsequently, due to respiratory insufficiency, the patient required intubation and sedation. Mechanical ventilation was started on synchronized intermittent mandatory ventilation mode with a tidal volume of 550 mL, positive end-expiratory pressure of 6 cm H_2_O, pressure trigger sensitivity of -2 cm H_2_O, fraction of inspired concentration of oxygen at 0.5, ventilatory rate of 16 breaths/min, and pressure support of 15 cm H_2_O. Tremors were a prominent finding in his upper extremities, and they also affected his face and lips (Video 1). Additional information about the patient’s history revealed that he had suffered from a mosquito bite-like rash 2 weeks before his presentation.

On the 5^th^ hospital day, lumbar puncture revealed clear and colorless cerebrospinal fluid without white blood cells, 36 mg/dL of glucose, and 106 mg/dL of protein. His blood test results were as follows: 10.3×10^3^/mL white blood cells, 73×10^3^/mL platelets, and 10.1 g/dL hemoglobin. His cranial computed tomography and magnetic resonance imaging demonstrated only atrophic changes, and his spinal magnetic resonance imaging did not reveal any abnormalities.

Routine cerebrospinal fluid and blood cultures, acid-fast bacillus testing, and herpes simplex virus polymerase chain reaction were negative. On the 5^th^ day of admission, his serum sample was sent to the National Arboviruses and Viral Zoonoses Reference Laboratory (Ankara, Turkey) for testing for arboviral infections. The West Nile virus real-time reverse transcriptase polymerase chain reaction (Anatolia Geneworks, İstanbul, Turkey) was positive, but indirect immunofluorescence test (Euroimmun, Luebeck, Germany) results were negative for both IgM and IgG. To demonstrate the seroconversion, a second serum sample was sent to the laboratory on the 9^th^ day, but anti-West Nile virus antibodies were still negative.

He underwent a course of plasmapheresis in the second week of his hospital stay. The plasmapheresis regimen consisted of the removal of -1 plasma volume during each cycle for a total of three cycles on an every other day basis. After undergoing plasmapheresis, the patient’s motor strength recovered from grade 0 to grade 2/5 in his upper extremities and from grade 0 to grade 1/5 in his lower extremities. Over the course of the next month, the patient showed partial and progressive improvement, especially in the upper extremities, through physical therapy. He regained his strength to a grade of 3/5 in his upper extremities. A third serum sample was positive for anti-West Nile virus-IgG on day 42.

The patient remained dependent on mechanical ventilation and developed ventilator-associated pneumonia due to multidrug-resistant *Acinetobacter baumannii* and renal failure requiring continuous venovenous hemodiafiltration. The patient did not improve and succumbed to his illness on day 184 after admission to the hospital.

## DISCUSSION

The symptoms of West Nile neuroinvasive disease include fever, headache, stupor, disorientation, coma, tremors, convulsions, muscle weakness, vision loss, numbness, and paralysis (2,3,4). Sejvar et al. (4) reported that the most common neurological finding was tremors that were detected at a rate of 94%. Similarly, Tilley et al. (5) reported that tremors are the most predictive neurological feature of West Nile virus infection. Previous immunohistochemical and neuroimaging studies have suggested that viral encephalitis-induced tremors and parkinsonism are caused due to abnormal changes in the basal ganglia, thalamus, and substantia nigra (3,6). The patient reported here presented with nausea and vomiting upon admission but later exhibited fever, muscle weakness, and prominent tremors. The patient’s tremor with a frequency of 4-5 Hz was prominent in the upper extremity and became apparent when he attempted to move his hand through the verbal command (Video 1). Unlike myoclonus, it was rhythmic and there was no jerk. It has earlier been reported that tremors associated with West Nile neuroinvasive disease involve primarily the upper extremities and are generally intentional or postural (4).

The differential diagnosis for acute flaccid paralysis includes, but is not limited to, West Nile neuroinvasive disease paralysis, Guillain-Barré syndrome, Lyme disease, heavy metal toxicity, botulism, myasthenia gravis, spinal cord compression, and poliomyelitis (7). Our initial diagnosis was West Nile neuroinvasive disease due to the patient’s rash history, known West Nile virus circulation in the geographical area, and prominent tremors. The laboratory diagnostic criteria for West Nile virus defined by the US Centers for Disease Control and Prevention include (i) isolation of the virus, specific viral antigens, or nucleic acids from the blood, tissues, cerebrospinal fluid, or other body fluids and (ii) detection of virus-specific IgM and/or IgG antibodies in serum or cerebrospinal fluid (2). A positive polymerase chain reaction test denotes a definitive diagnosis, but the viremia is extremely short and generally resolves by the time the symptoms begin unless the patient is immunosuppressed. Our patient was diagnosed using real-time reverse transcriptase polymerase chain reaction on the 5^th^ day of onset of the symptoms, which is indicative of a prolonged viremia. As the anti-West Nile virus IgG was found to be positive on the 42^nd^ day, the absence of anti-West Nile virus IgM on the 9^th^ day and the 42^nd^ day may be related to the patient’s immunocompromised status, performed plasmapheresis, or both. Therefore, in patients with immunocompromised status and prolonged viremia, detection of West Nile virus RNA in serum would be a more informative and reasonable diagnostic criterion than serological detection of West Nile virus-specific antibodies.

Rituximab causes B-cell death by targeting the surface protein CD20. Recovery of B cells begins 6-9 months after the completion of therapy and generally takes at least 12 months to resume to normal levels. B-cell depletion can be expected to result in a reduced to absent humoral responses to new antigens. Blunted humoral response from rituximab may be accompanied by an increase in neurotropic and opportunistic viral infections. Some fatal viral infections have also been reported after rituximab treatment, such as cytomegalovirus, varicella-zoster virus, hepatitis B virus, enterovirus, and West Nile neuroinvasive disease (8,9). Goates et al. (8) reported that rituximab not only predisposes patients to more severe West Nile neuroinvasive disease infection but also results in negative serological tests, thereby leading to delayed diagnosis.

Respiratory failure requiring long-term mechanical ventilation support and subsequent tracheostomy have been described with West Nile neuroinvasive disease. A high percentage of affected patients require intubation because of severely reduced level of consciousness, bulbar weakness, and diaphragmatic and intercostal muscle paralysis (4,10). According to a retrospective study of 32 patients, respiratory complications were the leading cause of death in West Nile neuroinvasive disease presenting with flaccid paralysis (10). Furthermore, the authors reported that successful extubation occurred after prolonged weaning periods (mean duration of intubation 66 days) and often multiple attempts of extubation and reintubation. In patients with prolonged mechanical ventilation and intensive care unit stay, complications include ventilator-associated pneumonia, aspiration, atelectasis, thromboembolic disease, contractures, and decubitus ulcers.

There is no specific treatment for West Nile neuroinvasive disease, and patients are managed with supportive care. Although different therapeutic approaches have been suggested, including plasmapheresis, intravenous immunoglobulin, and ribavirin, there is no evidence to support them. Our patient underwent plasmapheresis due to its accessibility at our center; however, due to his poor recovery, it remains unclear whether his outcome was affected by this intervention.

**Video 1.** The patient with new-onset tremors. Tremors are a very common symptom of West Nile neurological disease. The observed tremors were characterized in the patient as upper extremity intentional tremors with asymmetrical presentations. In addition to the new-onset tremors, rapidly progressive muscle weakness and low-grade fever accompanied the symptoms of the patient. This video was recorded on the 10^th^ day in his intensive care unit follow-up.

**(10.4274/balkanmedj.galenos.2019.2018.12.74.video.1)**
